# Chemical-defined medium supporting the expansion of human mesenchymal stem cells

**DOI:** 10.1186/s13287-020-01641-7

**Published:** 2020-03-19

**Authors:** Jianyong Xu, Wei Lian, Jieting Chen, Wenlei Li, Lingyun Li, Zhong Huang

**Affiliations:** 1 Guangdong Provincial Key Laboratory of Regional Immunity and Diseases, Department of Immunology, School of Medicine, Shenzhen University, Nanhai Avenue 3688, Shenzhen, 518060 Guangdong People’s Republic of China; 2 Department of Obstetrics, People’s Hospital of Baoan, Shenzhen, 518055 People’s Republic of China; 3 Department of Obstetrics, Women and Children Health Institute of Futian, Shenzhen, 518055 People’s Republic of China

**Keywords:** Mesenchymal stem cells, MSCs, Chemical-defined medium, NBVbe medium

## Abstract

**Objectives:**

Mesenchymal stem cells (MSCs) have been intensively investigated as to their therapeutic potentials. However, the full chemical-defined medium supporting the isolation and expansion of human MSCs has not been developed yet.

**Materials and methods:**

Here, we developed the full chemical-defined medium, NBVbe medium, via RNA sequencing, bioinformatic analysis, and growth factor screening.

**Results:**

The NBVbe medium contains N2B27 medium with the BSA (bovine serum albumin) replaced by the recombinant human albumin, bFGF (basic fibroblast growth factor), vitamin C, and EGF (epidermal growth factor). The NBVbe medium could support the isolation and expansion of human MSCs from the umbilical cords.

**Conclusions:**

The full chemical-defined medium supporting the isolation and expansion of human MSCs has been developed. This would be helpful for further optimization of the MSC medium, their clinical applications, and molecular characterization.

## Introduction

Mesenchymal stem cells (MSCs) are widely investigated because of their therapeutic potentials. In addition to their abilities to differentiate into multiple cell types, they could orchestrate the immune responses and modulate the tissue microenvironment, resulting in tissue regeneration [1–6]. Their supportive roles in regulating hematopoietic stem cell (HSC) homeostasis also highlight their therapeutic applications in HSC transplantations [7, 8].

Despite the intensive studies on their therapeutic applications, many basic questions remain unsolved, such as the cell identity and origin. Differing from other types of adult stem cells, MSCs could be isolated from many tissues, such as the bone marrow, adipose, and umbilical cord [1, 7]. Whether they have the same origin and function remains unclear. Furthermore, their specific cell surface markers have not been developed so far [9]. Indeed, their definition, function, and therapeutic effects need to be delineated precisely [10].

One of the major obstacles to solving these basic questions is that the full chemical-defined medium supporting the expansion of human MSCs is missing. It has been demonstrated that the human serum and platelet lysate (PL) could support the human MSCs’ expansion in vitro [11, 12]. And the human platelet lysate has been used as the standard culture medium for MSCs’ clinical applications [13, 14]. However, the batch variation of human PL preparation affects the phenotype and function of MSCs [15, 16]. Furthermore, the human platelet has limited resources. Thus, developing a full chemical-defined medium for human MSCs is critical for both basic biology study and also the clinical applications.

To overcome the inconsistent performance associated with the human platelet lysate, lots of efforts have been made to uncover the critical components of the platelet lysate which supports the MSCs’ expansion [17–24]. Unfortunately, it remains largely unclear what constituents mainly contribute to the MSCs’ proliferation. Here, we would provide an alternative strategy to uncover those critical factors for supporting MSCs’ expansion. And finally, we established the full chemical-defined medium supporting the isolation and expansion of the human MSCs.

## Materials and methods

### Isolation and expansion of human MSCs with serum-based medium

This study was approved by the ethics committee of Shenzhen University and followed the tenants of the Declaration of Helsinki. The human umbilical cords were collected in 10 min after the baby’s birth and the informed consents were obtained. The umbilical cords were stored in DMEM/High Glucose (Gibco, USA) with antibiotics (500 units/mL of penicillin and 500 μg/mL of streptomycin) on ice and delivered to the lab. They were minced into 1–3 mm^3^ fragments immediately and incubated in 1 mg/mL collagenase B (STEMCELL Technologies, diluted in DMEM/High Glucose) for 12 h at 37 °C. Then the isolated single cells of human MSCs were expanded in DMEM/High Glucose (Gibco, USA) plus 10% human serum (purified from the umbilical cord blood), 10 ng/mL bFGF (basic fibroblast growth factor, Peprotech), 50 μg/mL vitamin C (Sigma), and antibiotics (100 units/mL of penicillin and 100 μg/mL of streptomycin). The human MSCs were passaged with TrypLE (Thermo Scientific).

### Sample treatment and RNA sequencing

Human MSCs at passage 2 were plated onto p6 plates at the concentration of 5 × 10^4^ cells per well. Then they were incubated with DMEM/High Glucose (Thermo Scientific), 1% platelet lysate (Merk) in DMEM/High Glucose (Thermo Scientific) plus 2 U/mL heparin (Sigma), 2% platelet lysate in DMEM/High Glucose plus 2 U/mL heparin, or 5% platelet lysate in DMEM/High Glucose plus 2 U/mL heparin for 5 days. The RNA was extracted with Eastep® Super Total RNA Extraction Kit (Promega). The quality control, library construction, RNA sequencing, and bioinformatic analysis were performed in BGI (Beijing Genomics Institute). The PCA (principal component analysis) was performed by princomp and ggplot2 packages in R. The KEGG (Kyoto Encyclopedia of Genes and Genomes) pathway analysis was performed by phyper in R. The protein-protein interaction analysis was conducted by DIAMOND and STRING. The expression pattern analysis was performed by Mfuzz package in R. All the analyses were conducted with the online bioinformatic platform Dr. Tom (biosys.bgi.com/) provided by BGI.

### Medium components screening

For basal medium screening, the human MSCs at passage 2 were plated onto p12 plates at the concentration of 2 × 10^4^ cells per well. Then they were incubated with indicated mediums for 5 days and the cell counted with hemocytometer. The medium was refreshed every 2 days. The DMEM medium was DMEM/High Glucose (Thermo Scientific). The FBS medium was composed of DMEM/High Glucose plus 10% FBS (fetal bovine serum, Thermo Scientific). The N2B27 medium was composed of DMEM/High Glucose plus N2 (Thermo Scientific) and B27 (Thermo Scientific). The N2B27-VA medium was composed of DMEM/High Glucose plus N2 (Thermo Scientific) and B27 without vitamin A (Thermo Scientific). The NB medium was composed of DMEM/High Glucose plus NB mixture (Table 1). The 5% PL medium was composed of DMEM/High Glucose plus 5% platelet lysate (Merk) and 2 U/mL heparin (Sigma).

**Table 1:** Composition of the NB medium (× 50)

Biotin	125 μg/mL
DL-alpha-tocopherol acetate	50 μg/mL
DL-alpha-tocopherol	50 μg/mL
Human recombinant albumin	2 mg/mL
Catalase	125 μg/mL
Human recombinant insulin	410 μg/mL
Human transferrin (Holo)	5 mg/mL
Superoxide dismutase	375 U/mL
Corticosterone	1 μg/mL
d-Galactose	750 μg/mL
Ethanolamine	0.05 μL/mL
Glutathione (reduced)	50 μg/mL
l-Carnitine	100 μg/mL
Linoleic acid	50 μg/mL
Linolenic acid	50 μg/mL
Progesterone	640 ng/mL
Putrescine	1.61 mg/mL
Sodium selenite	900 ng/mL
T3 (triodo-I-thyronine)	100 ng/mL

For growth factor or chemical screening, the human MSCs at passage 2 were plated onto p12 plates at the concentration of 2 × 10^4^ cells per well. The growth factors or chemicals were added into the NB medium and incubated the cells for 5 days. The medium was refreshed every 2 days. The concentration was used as 2 μM for BIO (6-bromoindirubin-3′-oxime), 50 μg/mL for Vc (vitamin C), 10 ng/mL for OSM (oncostatin M), PDGF (platelet-derived growth factor)-AA, PDGF-AB, PDGF-BB, bFGF, HGF (hepatocyte growth factor), EGF (epidermal growth factor), IGF (insulin-like growth factor), BDNF (brain-derived neurotrophic factor), NGF (nerve growth factor), VEGFA (vascular endothelial growth factor A), TGFβ2 (transforming growth factor beta 2), IL (interleukin)-1β, IL-6, IL-7, CSF2 (colony-stimulating factor 2), CXCL1 (C-X-C motif chemokine ligand 1), CXCL2, CXCL5, CXCL6, CXCL8, CXCL10, CXCL12, CCL2 (C-C motif chemokine ligand 2), CCL7, CCL11, and CCL20 (all from Peprotech).

### Cell doubling time assessment

The human MSCs at passage 2 were plated onto p6 plates at the concentration of 5 × 10^4^ cells per well. Then they were incubated with FBS medium (DMEM/High Glucose plus 10% FBS), 5% PL medium (DMEM/High Glucose plus 5% platelet lysate and 2 U/mL heparin), or NBVbe medium (DMEM/High Glucose plus NB, 50 μg/mL Vc, 10 ng/mL bFGF and 10 ng/mL EGF), as separate parallel experiments. Cells were passaged with TrypLE when they reached 80–90% confluence and replated with 5 × 10^4^ cells per well in p6 plate for continuous expansion.

### Flow cytometry

The human MSCs, expanded with the NBVbe medium, were dissociated with TrypLE and re-suspended in PBS containing 5% BSA (bovine serum albumin, Sigma). They were incubated with the mouse anti-human antibody CD90-FITC, CD73-FITC, CD105-FITC, HLADR-FITC, CD45-FITC, CD34-FITC, CD19-FITC, CD11b-FITC, or IgG-FITC (all from BD Biosciences) for 30 min. Cells were analyzed with the BD ACCURI C6 PLUS machine after being washed with PBS two times. The data were further analyzed with the FlowJo software.

### Colony formation assay

The human MSCs were dissociated with TrypLE and diluted with the 5% PL or NBVbe medium at the concentration of 1 × 10^4^ cell/mL. Then the hanging drops were performed on non-adhesive petri-dishes with 20 μL per drop. Six hours later, the 5% PL or NBVbe medium was added to the dish and cultured for 7 days. Then the colonies were plated onto 0.1% gelatin-coated dishes for 1 day and stained with crystal violet solution (0.05% crystal violet, 1% formaldehyde, and 1% methanol in PBS) for 20 min.

### Human MSC differentiation and characterization

The human MSC differentiation and characterization were conducted with StemPro® Adipogenesis Differentiation Kit (Gibco), StemPro® Osteogenesis Differentiation Kit (Gibco), StemPro® Chondrogenesis Differentiation Kit (Gibco), and Human Mesenchymal Stem Cell Functional Identification Kit (R&D Systems), according to the instructions.

### Isolation and expansion of human MSCs with the NBVbe medium

The human umbilical cords were collected in 10 min after the baby’s birth and the informed consents were obtained. The umbilical cords were stored in DMEM/High Glucose (Gibco, USA) with antibiotics (500 units/mL of penicillin and 500 μg/mL of streptomycin) on ice and delivered to the lab. They were minced into 1–3 mm^3^ fragments immediately and incubated with indicated dissociation reagents according to the instructions. The dissociation reagents include TrypLE (Gibco), 0.05% Trypsin-EDTA (Gibco), Accutase (STEMCELL Technologies), GCD (Gentle Cell Dissociation Reagent, STEMCELL Technologies), SR (ReLeSR™, STEMCELL Technologies), CDB (Cell Dissociation Buffer, enzyme-free, Gibco), ACF (Animal Component-Free Cell Dissociation Kit, STEMCELL Technologies), Collagenase B (STEMCELL Technologies, diluted in DMEM/High Glucose), and DNase I (Sigma), Dispase II (Sigma). Then the isolated single cells of human MSCs were expanded in the NBVbe medium (DMEM/High Glucose plus NB, 50 μg/mL Vc, 10 ng/mL bFGF, and 10 ng/mL EGF). The human MSCs were expanded with TrypLE (Thermo Scientific).

### Statistical analysis

Data were analyzed using SPSS software for Windows (SPSS Inc.) and are shown as mean ± SEM (standard error of the mean). Student’s *t* test was used for two-group comparison and 1-way ANOVA for multiple-group comparison with normal data distribution, parametric test, and Tukey’s post hoc tests. A level of *P* < 0.05 was considered statistically significant.

## Results and discussion

Lots of efforts have been made to uncover the critical components of the platelet lysate for supporting the MSCs’ expansion [17–24]. Unfortunately, it remains largely unclear what constituents mainly contribute to the MSCs’ proliferation. To develop the full chemical-defined medium for supporting the proliferation of human MSCs, we investigated the critical regulators, which are responsible for the MSCs’ proliferation, through analyzing the responses of the MSCs to the platelet lysate via RNA sequencing and bioinformatic analysis, instead of analyzing the platelet lysate itself. Therefore, the transcriptome of the MSCs was analyzed after exposing to different concentrations of human platelet lysate.

**Fig. 1:**
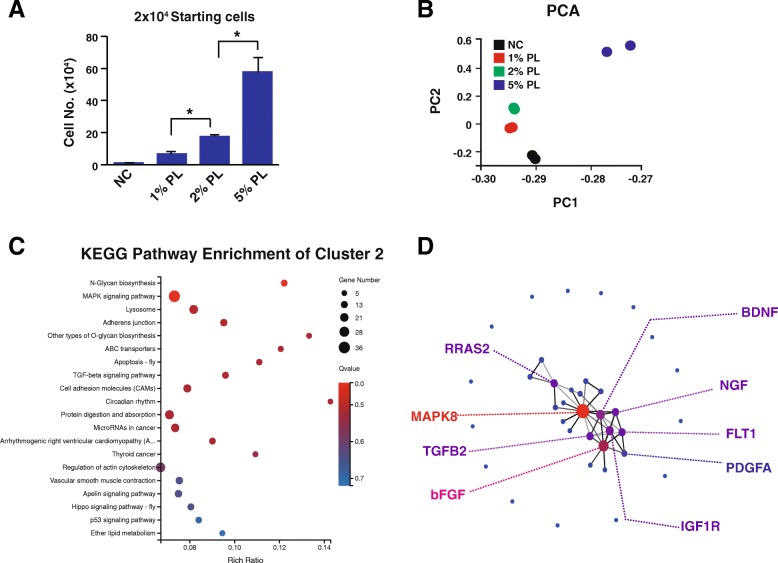
Transcriptome analysis of the human MSCs exposed to different concentrations of human platelet lysate. **a** Cell number counting after human MSCs exposed to 1%, 2%, and 5% PL in DMEM. Cells were plated onto p12 plates at 2 × 10^4^ cells per well and the cell number was counted 5 days later. **b** PCA of human MSCs exposed to different concentrations of human platelet lysate. **c** KEGG pathway enrichment of cluster 2 derived from the gene expression pattern clustering analysis. **d** Protein-protein interaction analysis of the genes derived from the MAPK signaling pathway of cluster 2. NC, negative control, Dulbecco’s modified Eagle’s medium-high glucose; PL, human platelet lysate; An asterisk indicates *P* < 0.05

The human platelet lysate had the dose effects on the proliferation of human MSCs (Fig. 1a). The PCA showed that the human MSCs treated with 1% and 2% platelet lysate had similar gene expression patterns while the 5% platelet lysate conferred human MSCs as a very different pattern (Fig. 1b). Therefore, the signal pathways or important genes responsible for human MSC proliferation should also have the dose effects. Gene expression pattern clustering among the samples treated without human platelet (negative control (NC)) or with different concentrations of human platelet lysate (1%, 2%, and 5% PL) was performed (Supplementary Fig. 1). Both upregulation and downregulation patterns were further analyzed, because the key components of the platelet lysate might have the positive regulations or negative feedback regulations on the gene expression of MSCs. Thus, the KEGG pathway enrichment analysis was performed for genes from clusters 2, 3, 5, 7, 8, 9, and 11 (Fig. 1c, Supplementary Fig. 2). Among all these important pathways activated or suppressed by the human PL, the MAPK (mitogen-activated protein kinase) signal pathway was further studied as it has been demonstrated as one key pathway for cell proliferation [25]. Protein-protein interaction network analysis showed that a panel of important growth factors or receptors was involved in the MAPK pathways which might contribute to the supportive role of PL for human MSC proliferation (Fig. 1d).

**Fig. 2:**
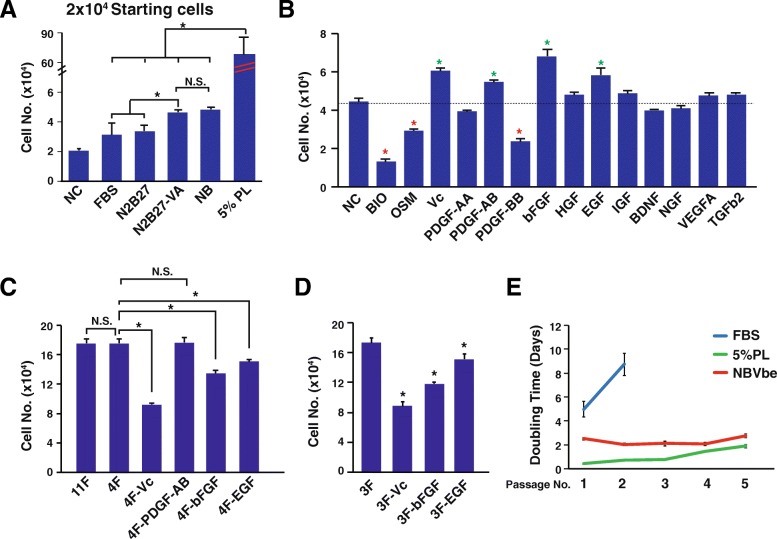
Full chemical-defined medium development. **a** Basal medium comparison. Human MSCs were plated onto p12 plates at 2 × 10^4^ cells per well and the cell number was counted 5 days later. **b**–**d** Growth factor and chemical screening. Human MSCs were plated onto p12 plates at 2 × 10^4^ cells per well and the cell number was counted 5 days later. **e** Cell doubling time analysis for the human MSCs cultured with the FBS, 5% PL, and NBVbe medium. The cells were continuously cultured for 5 passages. FBS. fetal bovine serum; NB, the N2B27-VA medium with the BSA (bovine serum albumin) replaced by the recombinant human albumin; PL, platelet lysate; BIO, 6-bromoindirubin-3′-oxime; OSM, oncostatin M; Vc, vitamin C; PDGF, platelet-derived growth factor; bFGF, basic fibroblast growth factor; HGF, hepatocyte growth factor; EGF, epidermal growth factor; IGF, insulin-like growth factor 1; BDNF, brain-derived neurotrophic factor; NGF, nerve growth factor; VEGFA, vascular endothelial growth factor A; TGFb2, transforming growth factor beta 2; 11F, Vc + PDGF-AA + PDGF-AB + bFGF + HGF + EGF + IGF + BDNF + NGF + VEGFA + TGFb2; 4F, Vc + PDGF-AB + bFGF + EGF; 3F, Vc + bFGF + EGF. An asterisk indicates *P* < 0.05; N.S. indicates non-significant

To analyze these potential important growth factors uncovered from the RNA-sequencing analysis, several basic mediums were firstly tested. Ideally, the basic medium should support the human MSCs alive or proliferation and also have the potential to develop as a full chemical-defined medium. The N2B27 medium is a serum-free medium suitable for neural stem cell and pluripotent stem cell proliferation [26, 27]. Thus, two types of N2B27 were compared, with or without vitamin A. Data showed that the N2B27-VA (N2B27 without vitamin A) could support the human MSC proliferation, much better than the N2B27 with vitamin A or FBS (fetal bovine serum) (Fig. 2a). Although the recipe of commercial N2B27 is unavailable to the public, the more original recipe and modified version of N2B27 had been published [28, 29]. Then the BSA (bovine serum albumin) component of the N2B27-VA was replaced by recombinant human albumin, termed as the NB medium (Table 1). Data showed that the NB medium was comparable to the N2B27-VA medium (Fig. 2A). Then 12 growth factors uncovered from the RNA-sequencing analysis were added to the NB medium to assess whether they could support the human MSC proliferation. Furthermore, we also tested two factors, BIO (6-bromoindirubin-3′-oxime, the GSK3β inhibitor) and OSM (oncostatin M) that were used for cardiac progenitor derivation which also expresses CD105 [30]. Totally, 14 growth factors or chemicals were evaluated. Among them, the Vc (vitamin C), PDGF-AB (platelet-derived growth factor AB), bFGF (basic fibroblast growth factor), and EGF (epidermal growth factor) showed beneficial effects on MSC proliferation while BIO, OSM, and PDGF-BB significantly suppressed their expansion (Fig. 2b). The four-factor combination (4F), including Vc, PDGF-AB, bFGF, and EGF, showed similar beneficial effects on MSC proliferation when compared with combining them together (the eleven-factor combination, excluding BIO, OSM, and PDGF-BB, 11F) (Fig. 2c). And further eliminating the PDGF-AB did not affect the MSCs proliferation while eliminating the Vc, bFGF or EGF significantly suppressed their proliferation (Fig. 2c). Thus, the 3F (3 basic factors), including Vc, bFGF, and EGF, is the minimal combination for supporting the human MSCs’ expansion, as eliminating anyone of them could significantly inhibit the cell proliferation (Fig. 2d). Although the NB medium plus 3F, termed as the NBVbe medium, had longer doubling time than the 5% PL, it was much better than the FBS-based medium (Fig. 2e).

**Fig. 3:**
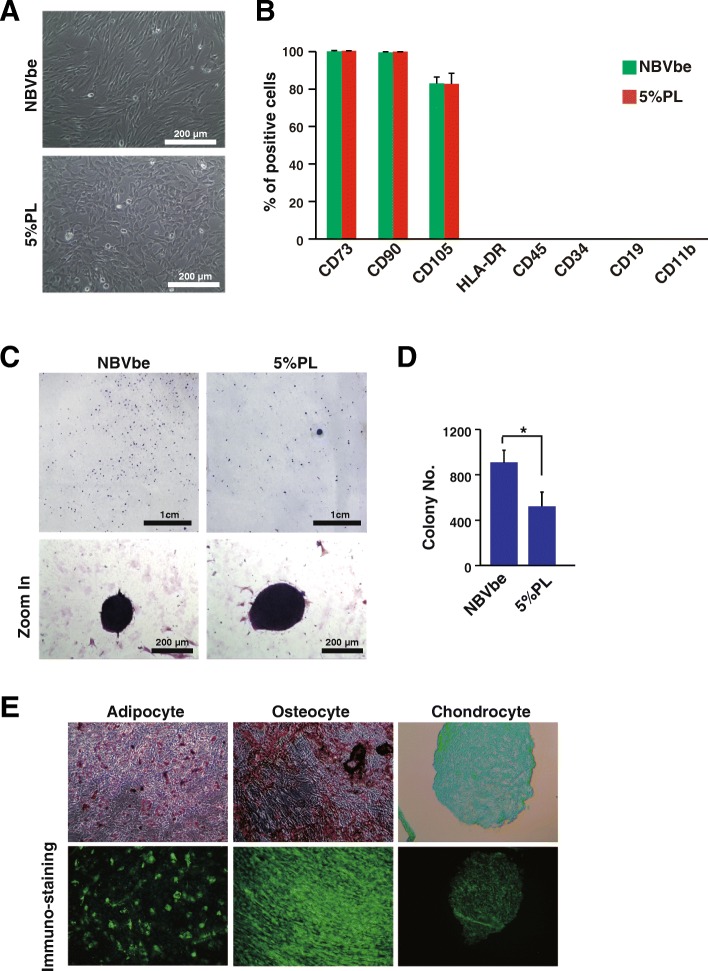
Characterization of human MSCs cultured with the NBVbe medium. **a** The human MSCs cultured with NBVbe medium had the different cell morphology from them cultured with the 5% PL medium. Scale bar, 100 μm. **b** Cell surface marker analysis by flow cytometry. **c** Colony formation assay for human MSCs cultured with the NBVbe medium or 5% PL medium. Scale bar for the upper panel, 1 cm. Scale bar for the lower panel, 200 μm. **d** Colony number counting for human MSCs cultured with the NBVbe medium or 5% PL medium for 5 passages. **e** The human MSCs were directed to adipocytes, osteocytes, and chondrocytes after cultured with the NBVbe medium for 5 passages. Left: Oil Red O staining and anti-FABP4 immuno-staining for the differentiated adipocytes; middle: Alizarin Red S staining and anti-Osteocalcin immunostaining for the differentiated osteocytes; right: Alcian blue staining and anti-Aggrecan immunostaining for the differentiated chondrocytes. Scale bar, 200 μm. An asterisk indicates *P* < 0.05

To further confirm that the NBVbe medium could really support the expansion of human MSCs, human MSCs were characterized after cultured with the NBVbe medium for 5 passages. They showed more fibroblast-like morphology (Fig. 3a) and were positive for MSC cell surface marker CD73, CD90, and CD105 while negative for HLA-DAR, CD45, CD34, CD19, and CD11b (Fig. 3b). The human MSCs cultured with the NBVbe medium could form more colonies than the platelet lysate medium (Fig. 3c, d) and differentiate into adipocytes, osteocytes, and chondrocytes (Fig. 3e).

**Fig. 4:**
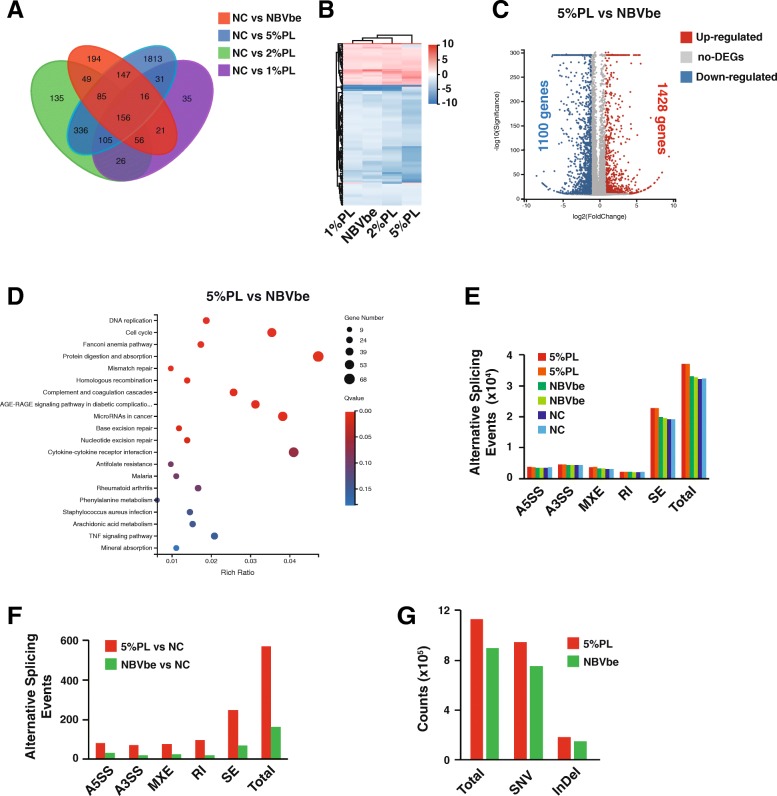
The RNA profile comparison between the human MSCs cultured with NBVbe or 5% PL. **a** Venn diagram showing the numbers of genes differentially regulated in human MSCs cultured with NBVbe, 1% PL, 2%PL, or 5% PL medium. **b** Hierarchical clustering analysis of mRNA profiles in human MSCs cultured with NBVbe, 1% PL, 2%PL, or 5% PL medium. **c** Differentially expressed genes between the human MSCs cultured with NBVbe and 5%PL. **d** KEGG pathway enrichment for differentially expressed genes between the human MSCs cultured with NBVbe and 5% PL. **e** Alternative splicing events detected by RNA sequencing. **f** Alternative splicing events compared with the human MSCs cultured with DMEM via RNA sequencing analysis. **g** SNV (single-nucleotide variant) and InDel (insertion and deletion mutations) detected by DNA exon sequencing. NC, negative control, human MSCs cultured with DMEM; DEGs, differentially expressed genes; A5SS, alternative 5′ splicing site; A3SS, alternative 3′ splicing site; MXE, mutually exclusive exons; RI, retained intron; SE, skipped exon

Because the MSCs cultured in the NBVbe medium were less proliferative than the PL medium, we were wondering whether further improvements could be made. RNA sequencing showed that the MSCs cultured with the NBVbe medium had a very different gene expression pattern when comparing them in culture medium containing 1%, 2%, or 5% PL (Fig. 4a). And gene clustering analysis indicated that the NBVbe medium might be more closed to the medium containing 1% platelet lysate (Fig. 4b). Indeed, there were still a large amount of differentially expressed genes between the MSCs cultured in the NBVbe medium and 5% PL (Fig. 4c). KEGG pathway enrichment analysis for these differentially expressed genes indicated that they were mostly involved into the cell cycle and DNA replication pathways (Fig. 4d). Protein-protein interaction network analysis uncovered 15 cytokines might support the proliferation of human MSCs (Supplementary Fig. 3A, 3B). Unfortunately, these cytokines could not further promote the MSCs’ proliferation in both the NB medium or the NB medium plus 3F (Supplementary Fig. 3C, 3D). Interestingly, the DNA repair pathway was also activated by the PL medium (Fig. 4d), which indicated that the 5% PL might induce genome instability, the adverse effects alongside the fast cell proliferation. RNA sequencing also revealed that the cells cultured with 5% PL had more alternative splicing events than those cells cultured with the NBVbe medium (Fig. 4e, f). Furthermore, the DNA exon sequencing revealed that the MSCs cultured with 5% PL might have more SNP (single-nucleotide polymorphism) events (Fig. 4g). Thus, the NBVbe medium might introduce less DNA mutations than PL.

**Fig. 5:**
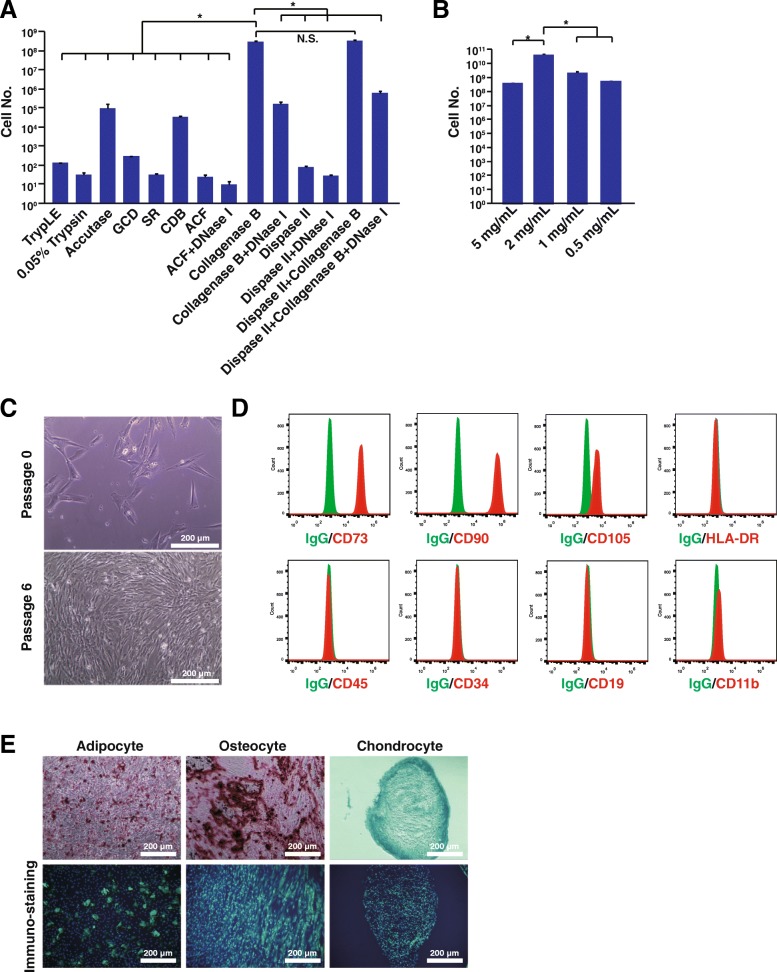
The NBVbe medium supports the human MSC isolation from the umbilical cords. **a** Cell isolation reagents comparison. Cell number was counted 30 days after the cell isolation. **b** Human MSCs were isolated with the different concentration of collagenase B. Cell number was counted 30 days after the cell isolation. **c** Human MSCs isolated from the umbilical cord, passage 0 and 6 cultured with the NBVbe medium. **d** Flow cytometry analysis of the human MSCs after isolated and cultured with the NBVbe medium. **e** Human MSCs were isolated and expanded with the NBVbe medium and then differentiated into adipocytes, osteocytes, and chondrocytes. Left: Oil Red O staining and anti- FABP4 immunostaining for the differentiated adipocytes; middle: Alizarin Red S staining and anti-Osteocalcin immunostaining for the differentiated osteocytes; right: Alcian blue staining and anti-Aggrecan immunostaining for the differentiated chondrocytes. Scale bar, 200 μm. An asterisk indicates *P* < 0.05. GCD, gentle cell dissociation reagent; SR, ReLeSR™; CDB, cell dissociation buffer, enzyme-free; ACF, animal component-free cell dissociation kit

Thus, the full chemical-defined medium for expanding the human MSCs was developed. Then we were wondering whether it also supports the isolation of MSCs from human umbilical cord tissues. Several dissociation enzymes, reagents, and different combinations were assessed to compare their efficiency on the isolation of human MSCs. Among them, the collagenase B was the most efficient enzyme for human MSC isolation from the umbilical cords in the NBVbe medium (Fig. 5a). Concentration optimization showed that the 2 mg/mL collagenase B was the most efficient concentration (Fig. 5B). The isolated human MSCs were fibroblast-like cells and could be expanded with the NBVbe medium (Fig. 5c). They were positive for CD73, CD90, and CD105 expression and negative for HLA-DR, CD45, CD34, CD19, and CD11b expression (Fig. 5d). Directed differentiation assay showed that these human MSCs could be differentiated into adipocytes, osteocytes, and chondrocytes (Fig. 5e). Therefore, the NBVbe medium could be used for the isolation of human MSCs from the umbilical cords.

In conclusion, we have developed the full chemical-defined medium for isolation and expansion of the human MSCs. The human MSCs cultured with the NBVbe medium had the self-renewal and differentiation abilities with expressing MSC markers, although the bFGF and vitamin C had been demonstrated as effective factors to support the human MSCs’ proliferation in the conventional medium [31–33]. The NBVbe medium we developed here is the first chemical-defined medium supporting the proliferation of human MSCs with the formulation available to the public. Moreover, this would be very helpful for molecular mechanism studies of the human MSCs, including the cell identity characterization. However, the NBVbe medium was not as good as the human platelet in supporting the proliferation of human MSCs. Thus, more effects should be made to further optimize the chemical-defined medium.

## Supplementary information

**Additional file 1: Figure S1.** The top 12 expression pattern clusters analyzed by Mfuzz package in R. (EPS 14642 kb)

**Additional file 2: Figure S2.** KEGG pathway enrichment of cluster 3, 5, 7, 8, 9 and 11 derived from the gene expression pattern clustering analysis. (EPS 1956 kb)

**Additional file 3: Figure S3. (A)** Protein-protein interaction analysis of the genes derived from the Cell cycle and DNA replication pathway of differentially expressed genes between the cells cultured with the NBVbe medium and 5% PL medium. **(B)** Protein-protein interaction analysis of the genes derived from the Cytokine and cytokine receptor interaction pathway of differentially expressed genes between the cells cultured with the NBVbe medium and 5% PL medium. **(C, D)** Growth factor and chemicals screening. Human MSCs were plated onto p12 plates at 2x10^4^ cells per well and the cell number was counted 5 days later. 3F: Vc + bFGF + EGF; NC: negative control, NB medium; IL: interleukin; CSF2: colony stimulating factor 2; CXCL: chemokine (C-X-C motif) ligand; CCL: chemokine (C-C motif) ligand. (EPS 1512 kb)

## Data Availability

All related data are available under request.

## Abbreviations

MSCs: Mesenchymal stem cells
BSA: Bovine serum albumin
HSCs: Hematopoietic stem cells
PL: Platelet lysate
NC: Negative control
MAPK: Mitogen-activated protein kinase
N2B27-VA: N2B27 without vitamin A
BIO: 6-Bromoindirubin-3′-oxime
OSM: Oncostatin M
Vc: Vitamin C
PDGF: Platelet-derived growth factor
bFGF: Basic fibroblast growth factor
HGF: Hepatocyte growth factor
EGF: Epidermal growth factor
IGF: Insulin-like growth factor 1
BDNF: Brain-derived neurotrophic factor
NGF: Nerve growth factor
VEGFA: Vascular endothelial growth factor A
TGFb2: Transforming growth factor beta 2
